# Assessment of cognitive function among adults aged ≥ 60 years using the Revised Hasegawa Dementia Scale: cross-sectional study, Lao People's Democratic Republic

**DOI:** 10.1186/s12961-022-00919-x

**Published:** 2022-11-29

**Authors:** Sengchanh Kounnavong, Manithong Vonglokham, Somphou Sayasone, Vanthanom Savathdy, Emiko Masaki, Ryoma Kayano, Bounfeng Phoummalaysith, Boungnong Boupha, Nobuyuki Hamajima

**Affiliations:** 1 Lao Tropical and Public Health Institute, Ministry of Health, Vientiane, Lao People’s Democratic Republic; 2 The World Bank, Vientiane, Lao People’s Democratic Republic; 3 Centre for Health Development, World Health Organization, Kobe, Japan; 4 Ministry of Health, Vientiane, Lao People’s Democratic Republic; 5 Department of Healthcare Administration, Nagoya University Graduate School of Medicine, Nagoya, Japan

**Keywords:** Dementia, Lao People’s Democratic Republic, Low- and middle-income countries, Health system strengthening, Population ageing

## Abstract

**Background:**

Rapid population ageing remains an important concern for health, social and economics systems; thus, a broader assessment of cognitive decline among adults aged ≥ 60 years is essential. It is important to regularly collect reliable data through validated and affordable methods from people living in different areas and in different circumstances to better understand the significance of this health problem. This study aimed to identify the prevalence of cognitive impairment and the related risk factors by reassessing the scoring of the Revised Hasegawa Dementia Scale among older adults in the Lao People’s Democratic Republic.

**Methods:**

A community-based cross-sectional investigation was conducted in rural and urban settings in six districts of three provinces in the country from January to July 2020. In total, 2206 individuals aged 60–98 years (1110 men and 1096 women) were interviewed in person using a pretested Lao version of the Revised Hasegawa Dementia Scale and the WHO STEPwise approach to noncommunicable disease (NCD) risk factor surveillance (the STEPS survey tool). The adjusted odds ratios (AORs) and 95% confidence intervals (95% CIs) were estimated using a logistic model.

**Results:**

The study found that 49.3% (1088/2206) of respondents (39.7% [441/1110] of men and 59.0% [647/1096] of women) had scores associated with some level of cognitive impairment. In addition to age, the following factors were significantly associated with cognitive impairment: having no formal education (AOR = 9.5; 95% CI: 5.4 to 16.8, relative to those with a university education), living in the northern region of the country (AOR = 1.4; 95% CI: 1.1 to 1.9, relative to living in the central region), living in a rural area (AOR = 1.5; 95% CI: 1.2 to 1.8), needing assistance with self-care (AOR = 1.8; 95% CI: 1.2 to 2.7) and being underweight (AOR = 1.5; 95% CI: 1.1 to 2.2). Factors associated with no cognitive impairment among older adults include engaging in moderate-intensity physical activity lasting for 10 minutes and up to 1 hour (AOR = 0.6; 95% CI: 0.5 to 0.8) and for > 1 hour (AOR = 0.6; 95% CI: 0.4 to 0.8).

**Conclusions:**

Using the Lao version of the Revised Hasegawa Dementia Scale, this study found that more than half of adults aged ≥ 60 years had cognitive impairment, and this impairment was associated with several risk factors. The limitations of this study may include possible overdetection due to the cutoff point for the assessment of cognitive decline used in the Revised Hasegawa Dementia Scale, given that the participants were not familiar with the instrument. However, the study results can be used to help inform health policy in the Lao People’s Democratic Republic regarding the urgent need for a routine data collection system and for providing an environment that addresses and reduces the identified risk factors for cognitive decline to mitigate their impact.

## Background

Ageing represents a risk factor for chronic diseases, including heart diseases, chronic obstructive pulmonary disease, diabetes, depression and dementia. Dementia is a syndrome, usually of a chronic or progressive nature, that leads to deterioration in cognitive function (i.e. the ability to process thoughts) beyond what might be expected from the usual consequences of biological ageing. While age is the strongest known risk factor for cognitive decline, dementia is not an inevitable consequence of ageing [1]. In 2020, more than 55 million people worldwide were living with dementia. This number will almost double every 20 years, reaching 78 million in 2030 [2]. Data can be collected to strengthen health information systems for health planning and to ensure that policies that aim to ensure universal health coverage consider older adults [3, 4].

Increases in the number of people with cognitive decline who need social and healthcare services have been observed not only in high-income countries but also in low- and middle-income countries [5]. Because there is a need for timely detection and for risk factors to be addressed to mitigate the impact of cognitive deterioration, it is essential to regularly collect reliable data through validated and affordable methods from people living in different areas and in different circumstances to better understand this health problem.

The Lao People's Democratic Republic (Lao PDR) is a country in South-East Asia with around 7 231 000 people, and in 2020, 7.1% of the population was aged ≥ 60 years [6]. The government of the Lao PDR aims to promote healthy ageing and ensure that older adults receive benefits as part of its goal of achieving universal health coverage, and it is introducing reforms to achieve comprehensive social protection for everyone by 2030 [7]. The adoption in 2012 of Prime Minister’s Decree 470 provided the legal framework for the establishment of the National Health Insurance Bureau and the integration of existing social health protection schemes into a single-payer system, under the management of the Ministry of Health and the National Health Insurance Bureau. By the end of 2018, the new National Health Insurance Bureau covered all 17 provinces, except the capital city. In 2019, a new health insurance law was announced [8] to eliminate financial barriers to accessing health services and maximize health benefits for everyone.

As of 2020, the public sector had five central hospitals, three specialist hospitals, 17 provincial hospitals, 135 district hospitals and 1070 health centres [6]. There are few private hospitals, and most private clinics are available only in urban areas. Currently, there are no public or private long-term care facilities for people with dementia, so care is usually provided by the family within the community.

The Lao PDR has committed to achieving the Sustainable Development Goals by 2030 [9]. There is a need to plan for change and for adaptation in light of the country’s demographic and health challenges. In recognition that the Lao population will be ageing and life expectancy will be prolonged (in 2020, it was 68.5 years), the implications for access to and equity in healthcare and for financial protection from healthcare costs for older people should be explicitly identified during each step of the process.

Cognitive function has not been widely investigated in the Lao PDR, and until recently, there was no nationally standardized method that could be used to measure it. Accordingly, the Revised Hasegawa Dementia Scale has been adapted for use in Lao PDR [10]. The Revised Hasegawa Dementia Scale is a simple questionnaire-based scale consisting of nine questions. The Revised Hasegawa Dementia Scale examines the main areas of cognitive function, including orientation, attention, language and memory. The Revised Hasegawa Dementia Scale questionnaire has been translated into Lao and validated through back-translation into English [10]. The Lao version of the Revised Hasegawa Dementia Scale was then used to assess the cognitive function of 414 older adults living in Vientiane Capital and Khammounane Province in 2017. This initial study revealed that a relatively high percentage (43.5%) of respondents had impaired cognitive function [11]. However, several factors can influence the Revised Hasegawa Dementia Scale score, and more detailed comparisons were needed to compare populations living in different areas and in different circumstances.

This study aimed to identify the prevalence of cognitive impairment and its risk factors by reassessing the scoring of the Revised Hasegawa Dementia Scale among older adults living in three regions (north, central and south) in the Lao PDR. Studies conducted in other countries have identified numerous sociodemographic, physical and mental conditions associated with cognitive impairment, including older age [12, 13], lower educational level [14, 15], gender [16, 17], tobacco smoking [18, 19], drinking alcohol [18, 20], having only a low level of activity [21, 22], being overweight or obese [23, 24], having hypertension [25] and having diabetes [26, 27]. Therefore, this study used the Revised Hasegawa Dementia Scale Lao version and the WHO STEPwise approach to noncommunicable disease (NCD) risk factor surveillance (STEPS) instrument (core and expanded) [28] to examine the prevalence and distribution of cognitive impairment in the Lao PDR and their relationship to older people's household characteristics, health and socioeconomic status.

## Methods

### Study area and participants

**Fig. 1:**
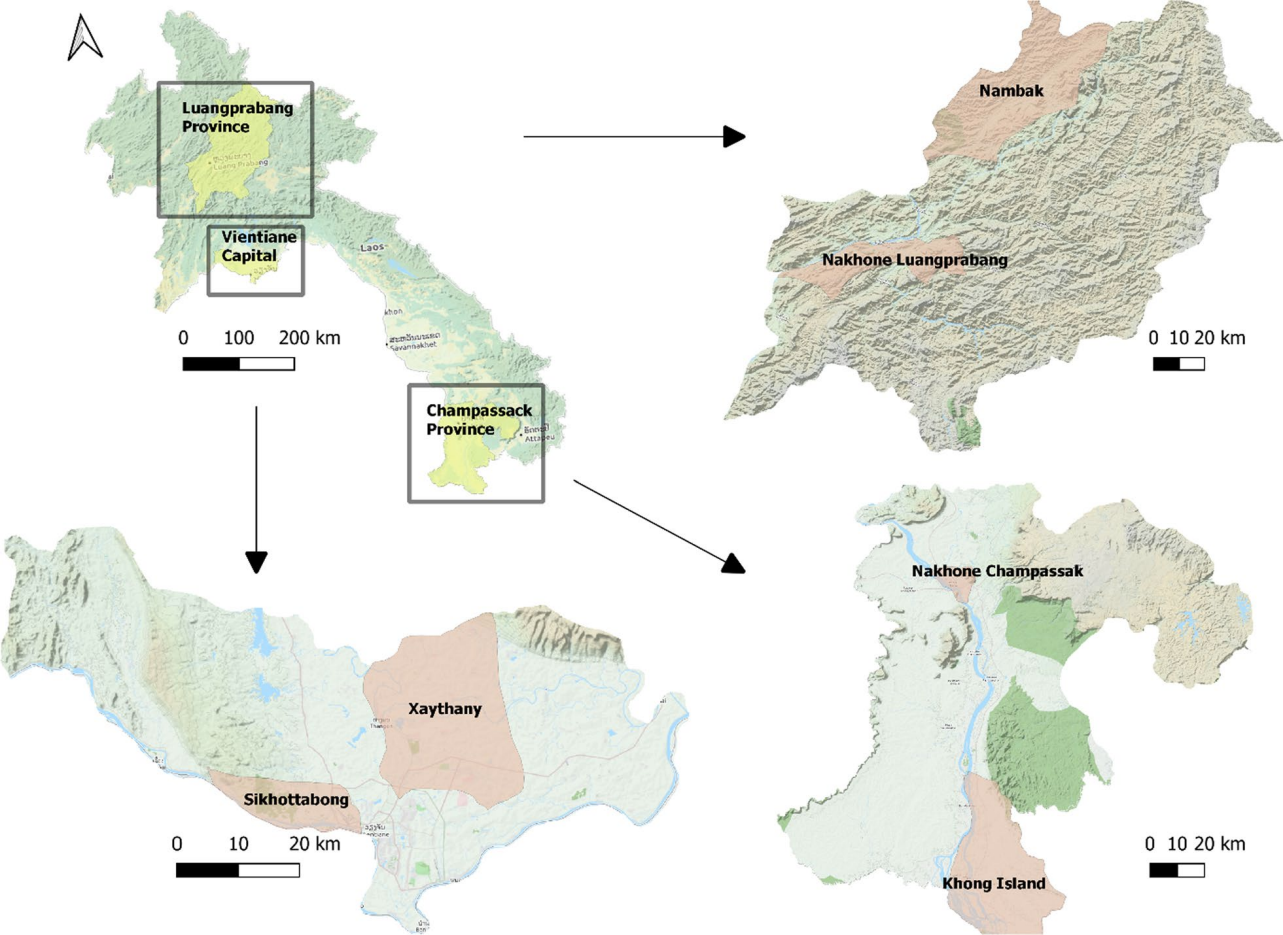
Map of the Lao PDR and the study sites

This community-based cross-sectional study surveyed adults aged ≥ 60 years who resided in six districts of three provinces in the Lao PDR: Luangprabang in the north, Vientiane Capital in the central region and Champassak in the south. In each province, two districts (one rural and one urban) were purposively selected: Nambak and Nakhone Luangprabang districts in Luangprabang Province, Sikhottabong and Xaythany districts in Vientiane Capital, and Nakhone Champassak and Khong Island districts in Champassack Province (Fig. 1). Simple random sampling was used for selecting target villages, households and the individual target population. Among a total of 81 villages, 13–14 villages per target district, and based on the family registry at local government authorities, we identified households where adults aged ≥ 60 years resided from each village, and we selected 30 households per village and at the household level; only one individual adult aged ≥ 60 years was selected.

All adults aged ≥ 60 years who had lived in the selected study areas for more than 6 months and consented to participate in the study were recruited. Those who did not understand the Lao language or had physical disorders or severe illness at the time of the interview were excluded. Although the total sample size required 2500 adults aged ≥ 60 years, 2320 subjects (92.8%) were recruited.

### Data collection

#### Interviews with heads of households with older adults

Face-to-face interviews were performed with heads of households using a tablet computer to record the answers. An identification and listing form and household information questionnaire were used to collect information about any older adults living in the household. The questionnaire was used to collect information related to demographic factors (age, gender, education, income, current job and type of current or last job) and about the main caregivers for older adults with low cognitive ability.

#### Interviews with the older adults

The WHO STEPS survey tool was used for the household survey. The STEPS survey tool is a standardized method for collecting, analysing and disseminating information about risk factors related to NCDs in WHO Member States [29]. The survey includes questions about health conditions (previously diagnosed diseases, health services received during the previous month and reason for the services, current medications, and membership in social health protection schemes) and lifestyle (alcohol drinking, smoking, use of chewing tobacco), physical activities and dietary habits.

#### Measuring cognitive function in adults aged ≥ 60

The Revised Hasegawa Dementia Scale Lao version questionnaire was delivered verbally during a face-to-face interview to assess the cognitive ability of the older adults living in the households. The Revised Hasegawa Dementia Scale is a screening tool for age-associated dementia that has a total score of 30 points. It consists of nine questions: question 1 on age (one point), 2 on temporal orientation (four points), 3 on spatial orientation (two points), 4 on registration (words) (i.e. repetition of three familiar words; three points), 5 on attention/calculation (i.e. subtracting 7 from 100 for twice; two points), 6 on counting backwards or backward repetition of three words and four digits (two points), 7 on recall of the three words memorized in question 4 (six points), 8 on registration (objects) (i.e. as confrontational naming and immediate recall of five objects; five points) and 9 on a category fluency test (i.e. word fluency; five points). The cutoff point of ≤ 20 was applied for dementia based on a study of the Revised Hasegawa Dementia Scale that reported this had 0.90 sensitivity and 0.82 specificity [29]. A score of ≤ 20 points is considered to be an indicator of cognitive impairment [30]. To accurately measure cognitive function, an Revised Hasegawa Dementia Scale manual previously developed [10] was used to standardize the skills of examiners.

#### Health checkup for participants aged ≥ 60 years

Weight, height, blood pressure and fasting blood glucose were measured for older adult participants at community venues, such as the village hall, school or temple. Weight was measured using a frequently calibrated electronic balance with 50 g sensitivity (Seca digital scale, Hamburg, Germany). Height was measured to 0.1-cm precision using a stadiometer. Body mass index (BMI) was calculated using weight divided by height squared (kg/m^2^). The Omron HEM-FM31 (Omron, Singapore) was used for blood pressure measurement. Capillary blood was taken from a finger for a blood glucose test.

Hypertension was defined as systolic blood pressure > 140 mm Hg or diastolic blood pressure > 90 mm Hg for the average of three measurements or treatment for raised blood pressure in the past 2 weeks. Diabetes mellitus was defined as fasting blood glucose > 126 mg/dL (3.9 mmol/L) or treatment with insulin or another medicine for high blood glucose, or both.

### Data management and statistical analysis

A data manager was assigned to oversee data management and the production of electronic records. The data manager's duties included coordinating data entry, reviewing data collection forms and ensuring the accurate and timely capture of data with the Dimagi CommCare programme (Dimagi, Cambridge, MA, USA) for tablet computers.

Electronic records were submitted to a central server. The data were retrieved from the Dimagi CommCare website and downloaded into an Excel spreadsheet, then checked for errors and cleaned. The cleaned data set was then transferred into Stata 16 for analysis (StataCorp, College Station, TX, USA). Continuous variables were expressed as the mean ± the standard deviation (SD), and categorical variables were expressed as numbers and percentages. The 95% confidence interval (CI) was calculated based on a binomial distribution. Cross-tabulations were conducted to assess the associations between two variables. Logistic regression analysis was applied to estimate the crude odds ratio (COR) and adjusted odds ratio (AOR) with their 95% CIs. Statistical significance was established at a *P* value of < 0.05. Only variables with a *P* value < 0.2 from the initial bivariate analysis were included in the multivariate analysis.

## Results

Table 1 presents the sociodemographic characteristics of the participants. Of the total 2320 participants recruited to this study, 114 participants were removed from the analysis due to poor hearing. Of the 2206 participants, 1110 (50.3%) were men, and 1096 (49.7%) were women, with a mean age of 67.7 years (SD = 6.0) for men and 69.1 years (SD = 6.8) for women. The majority (63.5%; 1401) of participants lived with their extended families; only 1.6% (35) lived alone (0.4% [9] of men and 1.2% [26] of women). Only 7.4% (163) were reported to need help with self-care. One third of the study population came from each of the three provinces, and half of participants lived in rural areas, and the other half in urban areas.

**Table 1:** Sociodemographic characteristics of study participants, Lao PDR

Characteristics	Total (*N* = 2206)		Men (*n* = 1110)		Women (*n* = 1096)	
	No.	%	No.	%	No.	%
Family characteristics						
Family type						
Living alone	35	1.6	9	0.4	26	1.2
Nuclear	770	34.9	386	34.7	384	35.0
Extended	1401	63.5	715	64.4	686	62.6
Needs support for self-care						
Yes	163	7.4	73	6.9	90	8.2
No	2043	92.6	1037	93.4	1006	91.8
Region						
North	700	31.7	353	31.8	347	31.6
Central	714	32.4	381	34.3	333	30.4
South	792	35.9	376	33.9	416	37.9
Area						
Rural	1081	49.0	564	50.8	517	47.2
Urban	1125	51.0	546	49.2	579	52.8
Ethno-linguistic group						
Lao-Tai	1959	88.0	975	87.8	984	89.8
Mon-Khmer	165	7.5	88	7.9	77	7.0
Chinese-Tibetan	8	0.4	3	0.3	5	0.5
Hmong-Mien	74	3.3	44	4.0	30	2.7
Individual characteristics						
Age (years)						
Mean age (SD)	68.4 (6.5)		67.7 (6.0)		69.1 (6.8)	
Age group						
60–64	731	33.1	409	56.0	322	44.0
65–69	655	29.7	339	51.8	316	49.2
70–74	387	17.5	189	48.8	198	83.2
75–79	256	11.6	99	38.7	157	125.8
≥ 80	177	8	74	41.8	103	58.2
Education level						
No formal education	655	29.7	138	12.4	517	47.2
Primary school	1161	52.6	661	59.5	500	45.6
Secondary school	258	11.7	202	18.2	56	5.1
University	132	5.9	109	9.8	23	2.1
Employment status						
Currently employed or working	1454	65.9	682	61.4	772	70.4
Unemployed, unable to work	217	9.8	129	11.6	88	8.0
Unemployed, able to work	309	14.0	179	16.1	130	11.9
Retired	226	10.2	120	10.8	106	9.7
Marital status						
Married	1362	61.7	975	87.8	387	35.3
Single	19	0.9	0	0	19	1.7
Separated/divorced/widowed	825	37.4	135	12.2	690	62.9

*SD* standard deviation

Nearly one third of respondents (33.1%; 731) were in the youngest age group (60–64 years): of these, 56.0% (409) were men and 44.0% (322) were women; 177 participants were aged ≥ 80 years (the oldest age group), accounting for 8.0% of participants, of whom 41.8% (74) were men and 58.2% (103) were women. More than half (52.6%; 1161) of the respondents had completed primary education. Among women, a higher percentage were more likely not to have had any formal education (47.2%; 517/1096), while among men, only 12.4% (138/1110) had not had any formal education. Only 5.9% (132) of all participants had attended university. The majority of participants (65.9%; 1454) reported they were currently working, and 61.7% (1362) were married.

Table 2 shows the substance-use behaviours and health-related characteristics of the respondents. Altogether, 32.0% (706) of respondents were current smokers: 53.0% (589/1110) of men and 10.7% (117/1196) of women. Among the 1110 men, 45.5% (505) were former drinkers, 38.0% (422) were occasional or social drinkers and 16.5% (183) were heavy drinkers. In terms of physical activity, 22.6% (251) of men engaged in vigorous-intensity workouts lasting ≥ 1 hour per day, 34.9% (387) engaged in moderate-intensity workouts lasting for ≥ 1 hour, 29.7% (330) walked or bicycled for > 1 hour, and 35.0% (389) were sedentary for ≥ 90 minutes. Among the 1096 women, 6.3% (69) engaged in vigorous-intensity workouts lasting ≥ 1 hour per day, 24.3% (266) engaged in moderate-intensity workouts lasting for ≥ 1 hour, 78.6% (861) walked or bicycled for at least 10 minutes and up to 1 hour, and 36.5% (400) were sedentary for ≥ 90 minutes.

**Table 2:** Description of substance-use behaviours and health-related characteristics of study participants, Lao PDR

Characteristics	Total (*N* = 2206)		Men (*n* = 1110)		Women (*n* = 1096)	
	No.	%	No.	%	No.	%
Substance-use behaviours						
Cigarette smoking						
Never smoker	1121	50.8	196	17.6	925	84.4
Former smoker	379	17.2	325	29.3	54	4.9
Current smoker	706	32.0	589	53.0	117	10.7
Alcohol drinking						
Lifetime abstainer/former drinker	1421	64.4	505	45.5	916	83.6
Occasional/social drinker	586	26.6	422	38.0	164	15.0
Heavy drinker	199	9.0	183	16.5	16	1.4
Physical activity (per day)						
Vigorous-intensity workout						
Never or < 10 minutes	1800	81.6	805	72.5	995	90.8
10 minutes to 1 hour	86	3.9	54	4.9	32	2.9
≥ 1 hour	320	14.5	251	22.6	69	6.3
Moderate-intensity workout						
Never or < 10 minutes	903	40.9	393	35.4	510	46.5
10 minutes to 1 hour	650	29.5	330	29.7	320	29.2
≥ 1 hour	653	29.6	387	34.9	266	24.3
Walking or bicycling for at least 10 minutes						
10 minutes to < 1 hour	1641	74.4	780	70.3	861	78.6
≥ 1 hour	565	25.6	330	29.7	235	21.4
Sedentary behaviour per day						
None or < 90 minutes	1417	64.2	721	64.9	696	63.5
≥ 90 minutes	789	35.8	389	35.0	400	36.5
Health-related characteristics						
Hypertension^a^						
No	1557	70.6	779	70.2	778	71.0
Yes	649	29.4	331	29.8	318	29.0
Diabetes mellitus^b^						
No	1764	79.9	916	82.5	848	77.4
Yes	442	20.0	194	17.5	248	22.6
Body mass index						
Underweight (< 18.5)	311	14.1	138	12.4	173	15.8
Normal (18.5–24.9)	1231	55.8	663	59.7	568	51.8
Overweight (25.0–29.9)	509	23.1	245	22.1	264	24.1
Obese (≥ 30)	155	7.0	64	5.8	91	8.3

^a^Hypertension was defined when a participant had systolic blood pressure > 140 mm Hg or diastolic blood pressure > 90 mm Hg for the average of three measurements or had been treated for raised blood pressure in the past 2 weeks

^b^Participants were defined as having diabetes mellitus when their fasting blood glucose was > 126 mg/dL (3.9 mmol/L) or they were taking insulin or other medicines to treat high blood glucose, or both

Among the men, 29.8% (331) had hypertension, and 17.5% (194) had diabetes mellitus. Among the women, 29.0% (318) had hypertension, and 22.6% (248) had diabetes mellitus. In assessing the BMI of the respondents, 14.1% (311) were underweight (12.4% [138] of men and 15.8% [173] of women), 23.1% (509) were overweight (22.1% [245] of men and 24.1% [264] of women) and 7.0% (155) were obese (5.8% [64] of men and 8.3% [91] of women).

Table 3 presents the scores for individual items on the Revised Hasegawa Dementia Scale by age group. The study found that 49.3% (1088/2206) of respondents (39.7% [441/1110] of men and 59.0% [647/1096] of women) had scores associated with some level of cognitive impairment. The mean score for all participants was 18.8 (SD = 5.4). The highest mean score was found in the group aged 60–64 years (mean = 20.4), followed by that in the group aged 65–69 years (mean = 19.6). The lowest mean score was found in the group aged ≥ 80 years (mean = 14.7). The mean scores for temporal orientation, spatial orientation, registration (words), attention/calculation, counting backwards, recall (words), registration (objects) and word fluency were highest among those in the group aged 60–64 years, followed by those in the group aged 65–69 year.

**Table 3:** Mean (SD) scores on the Revised Hasegawa Dementia Scale, by item and age group, Lao PDR

Question no. and category (no. of points)	Mean (SD) score					
	All participants (*N* = 2206)	Age group (years)				
		60–64 (*n* = 731)	65–69 (*n* = 655)	70–74 (*n* = 387)	75–79 (*n* = 256)	≥ 80 (*n* = 177)
1. Age (1)	0.9 (0.2)	1.0 (0.2)	1.0 (0.2)	1.0 (0.2)	0.9 (0.3)	0.9 (0.3)
2. Temporal orientation (4)	2.6 (1.4)	2.9 (1.3)	2.8 (1.3)	2.5 (1.4)	2.3 (1.4)	1.8 (1.4)
3. Spatial orientation (2)	1.9 (0.4)	1.9 (0.3)	1.9 (0.3)	1.8 (0.4)	1.8 (0.5)	1.7 (0.6)
4. Registration (words) (3)	2.8 (0.7)	2.8 (0.6)	2.8 (0.6)	2.8 (0.8)	2.7 (0.8)	2.5 (1.0)
5. Attention/calculation (2)	0.9 (0.8)	1.1 (0.7)	0.9 (0.7)	0.4 (0.7)	0.6 (0.7)	0.4 (0.6)
6. Counting backwards (2)	0.8 (0.8)	0.9 (0.7)	0.8 (0.7)	0.7 (0.7)	0.6 (0.7)	0.5 (0.7)
7. Recall (words) (6)	3.9 (2.1)	4.2 (2.0)	4.1 (2.0)	3.7 (2.1)	3.5 (2.1)	3.1 (2.2)
8. Registration (objects) (5)	3.9 (1.1)	4.1 (0.9)	4.0 (1.0)	3.7 (1.2)	3.6 (1.3)	3.1 (1.4)
9. Word fluency (5)	1.1 (1.3)	1.5 (1.4)	1.2 (1.3)	0.9 (1.1)	0.8 (1.2)	0.5 (1.0)
Total score (30)	18.8 (5.4)	20.4(5.4)	19.6(4.9)	17.8 (5.2)	16.8 (5.6)	14.7 (5.5)

The highest possible score is 30 points; a cutoff of ≤ 20 points was used to indicate cognitive impairment

*SD* standard deviation

Among the 2206 older adult participants, 163 (7.4%) reported that they needed help with self-care from a family member, and the primary caregiver was their daughter (42.9%; 70 participants), followed by their son (35.6%; 58), spouse (11.6%; 19) and others (9.2%; 16) (Table 4).

**Table 4:** Need for help with self-care among participants aged ≥ 60 years, by gender, Lao PDR

Characteristics	Total (*N* = 2206)		Men (*n* = 1110)		Women (*n* = 1069)	
	No.	%	No.	%	No.	%
Needs help with self-care	163	7.4	73	6.6	90	8.2
Primary caregiver						
Spouse	19	11.6	16	21.9	3	3.3
Daughter	70	42.9	29	39.7	41	45.6
Son	58	35.6	25	34.2	33	36.7
Other (paid caregiver)	16	9.2	3	4.0	13	14.4

Table 5 presents results of the multivariate logistic regression analysis for factors associated with cognitive impairment in both men and women. In the bivariate analysis, the following family characteristics were significantly associated with cognitive impairment: being an adult who needs assistance with self-care (COR = 3.4; 95% CI: 2.4 to 4.9), living in a rural area (COR = 1.8; 95% CI: 1.5 to 2.1) and being from the northern region (COR = 2.2; 95% CI: 1.7 to 2.7) or southern region (COR = 1.9; 95% CI: 1.6 to 2.4). The following individual factors were significantly related to impairment in cognitive function: being female (COR = 2.1; 95% CI: 1.8 to 2.6); being an older adult aged 65–69 years (COR = 1.6; 95% CI: 1.3 to 2.1), aged 70–74 years (COR = 2.7; 95% CI: 2.1 to 3.5), 75–79 years (COR = 3.6; 95% CI: 2.6 to 4.9), ≥ 80 years (COR = 8.7; 95% CI: 5.6 to 13.7); not having formal education (COR = 20.9; 95% CI: 11.4 to 38.2); being separated, divorced or widowed (COR = 2.1; 95% CI: 1.7 to 2.5); and being unemployed and unable to work (COR = 2.2; 95% CI: 1.6 to 3.0). For health-related and substance-use behaviours, the risk factors were being underweight (COR = 2.2; 95% CI: 1.7 to 2.9) and being sedentary (that is, sitting for > 90 minutes per day) (COR = 1.2; 95% CI: 1.0 to 1.3).

**Table 5:** Odds ratio and 95% confidence interval (95% CI) for risk factors for scoring ≤ 20 on the Revised Hasegawa Dementia Scale among adults aged ≥ 60 years, Lao PDR

Characteristics	Crude odds ratio (95% CI) with *P* value		Adjusted odds ratio (95% CI) with *P* value	
Family characteristics				
Family type				
Nuclear	1			
Extended	1.1 (0.9 to 1.3)			
Living alone	1.6 (0.8 to 3.2)			
Needs help with self-care				
No	1			
Yes	3.4 (2.4 to 4.9)	< 0.001	1.8 (1.2 to 2.7)	0.008
Area				
Urban	1			
Rural	1.8 (1.5 to 2.1)	< 0.001	1.5 (1.2 to 1.8)	< 0.001
Region				
Central	1			
North	2.2 (1.7 to 2.7)	< 0.001	1.4 (1.1 to 1.9)	0.014
South	1.9 (1.6 to 2.4)	< 0.001	1.2 (0.9 to 1.6)	0.121
Ethno-linguistic group				
Lao-Tai	1			
Mon-Khmer	2.4 (1.7 to 3.4)	< 0.001	1.5 (0.9 to 2.2)	0.093
Chinese-Tibetan	1.1 (0.3 to 4.5)	0.847	0.7 (0.2 to 3.6)	0.747
Hmong-Mien	4.5 (2.5 to 8.0)	< 0.001	2.4 (1.2 to 4.8)	0.011
Individual characteristics				
Gender				
Male	1			
Female	2.1 (1.8 to 2.6)	< 0.001	1.3 (0.9 to 1.8)	0.072
Age (years)				
60–64	1			
65–69	1.6 (1.3 to 2.1)	< 0.001	1.5 (1.2 to 1.9)	0.001
70–74	2.7 (2.1 to 3.5)	< 0.001	2.2 (1.6 to 3.0)	< 0.001
75–79	3.6 (2.6 to 4.9)	< 0.001	2.1 (1.5 to 3.0)	< 0.001
≥ 80	8.7 (5.6 to 13.7)	< 0.001	4.7 (2.9 to 7.6)	< 0.001
Education level				
University	1			
Secondary school	1.5 (0.8 to 2.6)	0.163	1.3 (0.7 to 2.3)	0.412
Primary school	4.6 (2.7 to 7.6)	< 0.001	3.0 (1.8 to 5.1)	< 0.001
No formal education	20.9 (11.4 to 38.2)	< 0.001	9.5 (5.4 to 16.8)	< 0.001
Marital status				
Married	1			
Single	1.5 (0.6 to 3.7)	0.375	0.9 (0.3 to 2.7)	0.923
Separated/divorced/widowed	2.1 (1.7 to 2.5)	< 0.001	1.2 (0.9 to 1.5)	0.111
Employment				
Currently employed or working	1			
Unemployed, unable to work	2.2 (1.6 to 3.0)	< 0.001	1.6 (1.1 to 2.2)	0.015
Unemployed, able to work	1.0 (0.8 to 1.3)	0.983	1.2 (0.9 to 1.5)	0.120
Retired	0.4 (0.3 to 0.6)	< 0.001	0.7 (0.5 to 0.9)	0.040
Physical activity				
Vigorous intensity workout (per day)				
Never or < 10 minutes	1			
10 minutes to 1 hour	0.9 (0.6 to 1.4)	0.658		
≥ 1 hour	0.8 (0.6 to 1.0)	0.113		
Moderate-intensity workout				
Never or < 10 minutes	1			
10 minutes to 1 hour	0.5 (0.4 to 0.6)	< 0.001	0.6 (0.5 to 0.8)	0.001
≥ 1 hour	0.4 (0.3 to 0.5)	< 0.001	0.6 (0.4 to 0.8)	< 0.001
Walking or bicycling				
Never or < 1 hour	1			
≥ 1 hour	0.5 (0.4 to 0.6)	< 0.001	0.9 (0.6 to 1.2)	0.737
Sedentary behaviour				
None or < 90 minutes	1			
≥ 90 minutes	1.2 (1.0 to 1.3)	0.112	0.8 (0.6 to 1.0)	0.182
Substance-use and health-related behaviours				
Cigarette smoking				
Never smoker	1			
Former smoker	0.6 (0.4 to 0.7)	< 0.001	0.9 (0.7 to 1.3)	0.967
Current smoker	0.7 (0.6 to 0.8)	< 0.001	1.2 (0.6 to 1.9)	0.183
Alcohol drinking				
Lifetime abstainer/former drinker	1			
Occasional/social drinker	0.5 (0.4 to 0.6)	< 0.001	0.8 (0.6 to 1.0)	0.131
Heavy drinker	0.4 (0.3 to 0.5)	< 0.001	0.7 (0.5 to 1.1)	0.193
Hypertension				
No	1		1	
Yes	0.7 (0.6 to 0.9)	0.006	1.0 (0.8 to 1.2)	0.892
Diabetes mellitus				
No	1			
Yes	1.1 (0.9 to 1.3)	0.338		
Body mass index				
Normal (18.5–24.9)	1			
Underweight (< 18.5)	2.2 (1.7 to 2.9)	< 0.001	1.5 (1.1 to 2.2)	0.012
Overweight (25.0–29.9)	0.6 (0.5 to 0.8)	< 0.001	0.7 (0.5 to 0.9)	0.026
Obese (≥ 30)	0.5 (0.4 to 0.8)	< 0.001	0.7 (0.5 to 1.1)	0.154

*CI* confidence interval

The following protective factors were identified: being retired from work (COR = 0.4; 95% CI: 0.3 to 0.6), engaging in moderate physical activity lasting for 10 minutes and up to 1 hour (COR = 0.5; 95% CI: 0.4 to 0.6) and lasting for > 1 hour (COR = 0.4; 95% CI: 0.3 to 0.5), walking or bicycling for > 1 hour (COR = 0.5; 95% CI: 0.4 to 0.6), being a former smoker (COR = 0.6; 95% CI: 0.4 to 0.7) and being an occasional or social drinker (COR = 0.5; 95% CI: 0.4 to 0.6).

In the adjusted analysis, the significant risk factors for cognitive impairment were being an adult who needs assistance with self-care (AOR = 1.8; 95% CI: 1.2 to 2.7); living in a rural area (AOR = 1.5; 95% CI: 1.2 to 1.8); being from the northern region (AOR = 1.4; 95% CI: 1.1 to 1.9); being aged 70–74 years (AOR = 2.2; 95% CI: 1.6 to 3.0), 75–79 years (AOR = 2.1; 95% CI: 1.5 to 3.0) and ≥ 80 years (AOR = 4.7; 95% CI: 2.9 to 7.6); having no formal education (AOR = 9.5; 95% CI: 5.4 to 16.8); and being underweight (AOR = 1.5; 95% CI: 1.1 to 2.2). However, participants were less likely to have cognitive impairment if they engaged in moderate-intensity physical activity lasting for 10 minutes and up to 1 hour (AOR = 0.6; 95% CI: 0.5 to 0.8) and for > 1 hour (AOR = 0.6; 95%: 0.4 to 0.8).

## Discussion

This study examined the proportion of adults aged ≥ 60 years with impaired cognitive function living in three different areas of the Lao PDR, using the Revised Hasegawa Dementia Scale Lao version. The study revealed that cognitive impairment was associated with age, particularly in respondents aged ≥ 65 years, and educational levels. Living in rural areas and in the northern region were associated with higher risk of cognitive impairment in comparison with those living in urban areas or the southern and central regions.

The lower Revised Hasegawa Dementia Scale scores in the current study were partly due to low scores on questions 5 and 6, which involve serial subtraction and counting backwards. Almost all respondents (81.3%) reported having had no formal or primary education. The low level of education among study participants might help explain the difficulties with questions 5 and 6. This study also found lower scores related to remembering words and confrontational naming (questions 7 and 8), and the lowest score was related to word fluency (question 9); these scores may reflect limited educational opportunities during adolescence. Many studies have suggested that having a higher level of education is a protective factor against developing cognitive impairment, indicating that attaining only a low education level is associated with poor cognitive function [15, 31]. Continual mental stimulation gained through learning may increase favourable structural or neurochemical alterations in the brain, thus improving cognitive function [32]. However, several cohort studies have not found associations between low education levels and cognitive decline [33, 34]. Adults aged ≥ 60 years in this study spent their adolescence in the middle of a civil war; therefore, the majority of them did not have any formal education.

Several studies have noted that women are more likely than men to develop cognitive impairment [16, 17]. We found that more women had developed cognitive impairment than men in the bivariate analysis but not in the multivariate analysis. In the Lao PDR, women tend to have a longer life expectancy, and this longer life will be healthier if preventive strategies are undertaken, such as using a multifocal approach to prevent and slow cognitive decline among older adults by encouraging exercise, avoidance of high levels of alcohol consumption, socializing and by preventing NCDs [18]. Additionally, the WHO global action plan on dementia recommends increasing public awareness of and developing programmes to encourage positive attitudes towards dementia in the community [35]. Without a strong health systems response and a formal long-term care sector, those who are disabled at older ages become the responsibility of family caregivers, such as daughters and spouses. Other studies have indicated that older adults who are divorced, separated or widowed have a higher risk of cognitive impairment [36], but this was observed only in the bivariate analysis in this study.

In the Lao PDR, the high prevalence of harmful alcohol use in adults aged ≥ 18 years [37] and chronic malnutrition during childhood might affect cognitive function [31, 38]. Risk factors for cognitive impairment include age, family history, education level, brain injury, exposure to pesticides or toxins, and physical inactivity.

Ageing is a risk factor for NCDs such as hypertension [25] and type 2 diabetes mellitus [26, 27]. In our study, more than one third of respondents had hypertension, and nearly one fifth had type 2 diabetes mellitus. Hypertension and diabetes mellitus account for both small and large vascular changes that can lead to cerebrovascular accidents, strokes, cerebral haemorrhage and micro-cerebral infarcts [39, 40]. Therefore, effective and timely preventive measures or strategies to address the risks of ill health are required. For instance, Japan has invested in primary, secondary and tertiary prevention strategies, especially those addressing hypertension and diabetes, and therefore has both a high life expectancy and a high healthy life expectancy, even among people in older age groups. Japan's health investments were initiated in the 1960s when it was a lower-income country and its population was relatively young. Japan continues to have the highest life expectancy globally, partly due to this early investment and also because people can continue to be active and healthy in their older years.

Although respondents who were overweight or obese were observed to have a lower risk of developing cognitive impairment in this study, many other studies have found that maintaining normal body weight throughout the life span is protective against cognitive impairment [25, 26].

Similar to other studies, respondents who engaged in moderate-intensity physical activity had a lower risk of cognitive impairment [14, 18, 21, 41].

One third of respondents were current smokers and current drinkers. Chronic smokers were more likely to drink alcohol, and smoking tobacco is associated with cognitive decline and neurocognitive diseases in later life [19]. However, this study did not find such an association.

In recent decades, the Lao PDR has transitioned from a low-income country to one that is lower-middle-income. When most households lived in poverty and few people had access to health services, many people died young due to poor nutrition and infectious diseases. Impoverished adults who suffered from poor nutrition and a lack of cognitive stimulation during their early life could perhaps be at risk of cognitive impairment. As poverty declined and health services improved in the Lao PDR, there was a rapid reduction in deaths caused by diseases such as malaria, tuberculosis and diarrhoea, with corresponding increases in life expectancy. However, infants may still be malnourished, and children may still not receive an education [38], both of which may impact development at an early stage and place them at risk of cognitive impairment in later life. In addition, unhealthy lifestyles in adult life, such as poor diet, smoking, consumption of alcohol and working in stressful occupations, increase the risks of NCDs such as obesity, diabetes, vascular diseases and hypertension, all of which are associated with cognitive impairment later in life [40, 41].

A limitation of this cross-sectional study is that it provides only a snapshot in time and does not provide information on cause and effect relationships between cognitive impairment and different risk factors. In addition, the cutoff Revised Hasegawa Dementia Scale score of ≤ 20 may not be appropriate for those unfamiliar with subtraction. Screening for cognitive impairment with the Revised Hasegawa Dementia Scale Lao version might be better if the cutoff was ≤ 18 and if question 5 was removed; the appropriateness of making these changes and using a maximum score of 28 (rather than 30) should be confirmed by comparing these scores with clinical diagnoses in future studies. Future research should be performed not only to detect general cognitive impairment but also to differentiate among specific types of cognitive impairment.

However, this study also has strengths: it was conducted in three different parts of the country and had a large sample of respondents representing urban and rural areas and different sociodemographic features. The study provides evidence for future policy planning in the Lao PDR as increasing numbers of older people will place greater demands on the health services, particularly for the management of chronic NCDs, such as hypertension and diabetes, which can contribute to cognitive decline. Older adults are often likely to have one or more of these conditions and require support from various professionals, such as physiotherapists, dietitians and mental health professionals. WHO and other agencies have long advocated for developing a people-centred, multidisciplinary approach to healthcare. Implementing a robust primary care system requires shifting investments in infrastructure and deploying staff to primary care facilities and health networks, and determining the appropriate technology to be used at all levels of the health system, in keeping with clinical and referral pathways. The critical questions are whether the Lao PDR is ready for such profound changes and what the implications of these changes are for health and social services [5, 42]. To address these questions, it is necessary to understand the public policies and disease prevention strategies that enable healthy ageing in order to focus on the opportunity to plan for universal health coverage in recognition that health systems of the future need to respond to older populations [1, 5] as part of the global commitments to the Sustainable Development Goals [9].

## Conclusions

In the current study, more than half of the respondents who were aged ≥ 60 years had some cognitive impairment, and being older than 65 years, having a low educational level and needing assistance with self-care were all associated with being at a higher risk of developing cognitive impairment. The limitations of this study may include possible overdetection due to the cutoff point for the assessment of cognitive decline used in the Revised Hasegawa Dementia Scale, given that the study participants were not familiar with the subtraction. However, the study results can be used to help inform health policy in the Lao PDR regarding the urgent need for a routine data collection system and for providing an environment that addresses and reduces the identified risk factors for cognitive decline to mitigate their impact. Primary healthcare workers could screen patients for cognitive impairment using the Revised Hasegawa Dementia Scale Lao version even in resource-constrained settings.

Along with the rise in obesity and increase in NCDs associated with recent economic growth and subsequent changes in socioeconomic status, the prevalence of cognitive impairment and subsequent dementia may well increase in the future. Instead of focusing only on medical services and healthcare at an individual level, policy planning needs to address the substantial determinants of poor health outcomes across the life course and work to create a healthy environment for every community, including ageing populations, now and in the future.

## Data Availability

The data sets used and analysed for this study are available from the corresponding author on reasonable request.

## Abbreviations

AORs: Adjusted odds ratios
BMI: Body mass index
CIs: Confidence intervals
COR: Crude odds ratio
Lao PDR: Lao People's Democratic Republic
SD: Standard deviation
